# Exploring Nursing Students' Perspectives on Patient Safety Culture in Clinical Settings: A Mixed‐Method Study

**DOI:** 10.1111/jocn.17812

**Published:** 2025-05-06

**Authors:** Daniela Bartoníčková, Dominika Kohanová, Simona Dobešová Cakirpaloglu, Alison Steven, Katarína Žiaková

**Affiliations:** ^1^ Department of Nursing, Faculty of Health Sciences Palacký University in Olomouc Olomouc Czech Republic; ^2^ Department of Nursing, Jessenius Faculty of Medicine in Martin Comenius University in Bratislava Bratislava Slovak Republic; ^3^ Department of Nursing, Faculty of Social Sciences and Health Care Constantine the Philosopher University in Nitra Nitra Slovakia; ^4^ Department of Humanities and Social Sciences Palacký University in Olomouc Olomouc Czech Republic; ^5^ Department of Nursing, Midwifery and Health Faculty of Health and Life Sciences, Northumbria University Newcastle Newcastle upon Tyne UK

**Keywords:** clinical setting, experience, mixed‐method study, nursing student, patient safety

## Abstract

**Background:**

Patient safety in undergraduate nursing studies is an indispensable component of the curriculum. The process of experiential learning from practice is of high value not only in terms of personal development but also enables students to identify and address critical areas of patient safety that require improvement.

**Aim:**

To explore Czech undergraduate nursing students' perceptions of patient safety culture during clinical practice through a mixed‐method sequential study.

**Methods:**

Data were collected between 2021 and 2024 using a mixed‐method approach. The quantitative phase utilised the hospital survey on patient safety culture for nursing students. Four hundred and eighty‐two undergraduate nursing students from 16 faculties across the Czech Republic participated. The subsequent qualitative phase employed semi‐structured interviews with 12 undergraduate nursing students from one faculty in the Czech Republic. Descriptive and inferential statistical methods were used to analyse quantitative results, complemented by a reflective thematic analysis of qualitative data.

**Results:**

The most negatively rated survey dimensions were ‘Frequency of events reported’ (37.0%) and ‘Nonpunitive responses to errors’ (42.4%). Predictors for reporting adverse events in clinical practice were ‘Indicators of good practice’ (*p* ≤ 0.05). Based on the quantitative phase, the interpretive journey of nursing students' experiences from Exposure to adverse events, through Feeling disconnected and Cognitive dissonance, to the necessity of Speaking up for patient safety culture was captured in the qualitative phase.

**Conclusions:**

Nursing students struggle to engage in a patient safety culture, particularly in reporting adverse events during clinical practice. Strengthening education on reporting and standards is essential for students, along with professional development for clinical staff to align practices and cultures.

**Summary**

- What is already known about this topic
  ○ Patient safety is an essential part of undergraduate nursing education.
  ○ Nursing students often experience barriers to engaging with a culture of safety during clinical practice.

- What this paper adds
  ○ Identifies fear of blame and underreporting of adverse events as key concerns among Czech nursing students.
  ○ Explores how emotional and cognitive dissonance may inhibit students from actively participating in patient safety practices.
  ○ Demonstrates the need for better alignment between clinical practice environments and educational expectations regarding safety culture.

- Implications for clinical practice and nursing education
  ○ Calls for stronger integration of reporting and safety standards into undergraduate nursing curricula.
  ○ Supports targeted training for clinical staff to foster an environment where nursing students feel safe to report and reflect on adverse events.
  ○ Suggests the incorporation of reflective learning strategies to empower students to speak up and contribute to a culture of safety.

## 1 Introduction

Patient safety is a global priority (World Health Organisation 2021), defined as ‘*a discipline in the health care professions that applies safety science methods toward the goal of achieving a trustworthy system of health care delivery’* (Emanuel et al. 2008, 6). A vision of healthcare facilities having a ‘culture of safety’ that can be measured, understood and improved has become a fundamental indicator of patient safety around the world (Nieva et al. 2003; World Health Organization 2021). In hospitals, patient safety culture (PSC) instruments have been developed to help identify areas requiring improvement, evaluate patient safety interventions or programmes and track changes over time (Reis et al. 2018). Additionally, qualitative syntheses have emphasised the need to assess PSC in a manner sensitive to hospital environments, organisational hierarchies and even national cultures (Alqattan et al. 2019; Tear et al. 2020). This is critical, as research highlights the pivotal role of nurses and other frontline staff in fostering a positive safety culture (Ammouri et al. 2015; de Lima Neto et al. 2017).

Nurses are the largest group of healthcare workers, estimated at 27.9 million worldwide (Buchan et al. 2022). However, even before the COVID‐19 pandemic, a global shortage of approximately 5.9 million nurses had already been identified. The pandemic's impact on workforce numbers is still unfolding, and compounding this challenge is the expectation that one in six nurses will retire within the next decade (ibid). This nursing workforce shortfall has implications for patient safety (Zaranko et al. 2023).

A systematic review by Alanazi et al. (2022) indicated that where nurses demonstrated positive attitudes toward patient safety, there were lower estimated rates of patient falls, medication errors, pressure ulcers, healthcare‐associated infections, use of physical restraints and adverse events, along with higher patient satisfaction. The RN4CAST (Nurse Forecasting: Human Resources Planning in Nursing) consortium and other researchers have reported that factors such as nurse staffing levels, skill mix, workload and burnout, work environment and nursing education significantly impact patient safety outcomes (e.g., Zaranko et al. 2023; Griffiths and Dall'Ora 2023; Dall'Ora et al. 2022; Griffiths et al. 2019). Nursing education programmes with a stronger emphasis on patient safety in their curricula have been associated with increased clinical competencies and a stronger focus on patient safety practices (e.g., Pearson and Steven 2018; Kirwan et al. 2019), contributing to improved patient safety outcomes. Thus, education can be considered an important starting point for patient safety empowerment (Pearson and Steven 2018; Steven et al. 2014; Mansour et al. 2012; Kirwan et al. 2019). Recent studies further support this, highlighting the positive impact of targeted patient safety education interventions on fostering a culture of safety among healthcare professionals (Agbar et al. 2023). Moreover, longitudinal research has shown that nursing students' knowledge and competencies in patient safety develop significantly over the course of their academic programmes, underlining the critical role of structured education in this area (Bressan et al. 2021).

## 2 Background

In 2009, the Council of the European Union recommended embedding patient safety in the education of all healthcare professionals (Kirwan et al. 2019). However, more than a decade later, European nursing programmes continue to face challenges in fully implementing this recommendation, largely due to persistent difficulties in integrating patient safety into curricula (Dissanayake et al. 2024; Kirwan et al. 2019). A recent literature review reinforced the need for patient safety education, emphasising system‐based components and fostering a culture of safety among students (Bedgood and Mellot 2021).

A study across 27 European countries found that patient safety is often not explicitly included in nursing curricula (Kirwan et al. 2019). While topics such as infection control, medication administration, transfusions, fall prevention (Kirwan et al. 2019; Lee et al. 2020) and occupational health (Boucaut and Cusack 2016) are covered within a patient safety framework, critical sociocultural aspects remain insufficiently addressed (Kirwan et al. 2019; Lee et al. 2020; Lukewich et al. 2015). This gap persists despite established recommendations and frameworks, including the National Patient Safety Education Framework (Australian Commission on Safety and Quality in Health Care 2005), the European Network for Patient Safety (European Union Network for Patient Safety 2010) and The Patient Safety Competency Framework for Nursing Students (Levett‐Jones et al. 2017).

Given that clinical experience accounts for approximately half of EU nursing curriculum hours, authors have highlighted the importance of focusing on student experiences of PSCs within practice learning settings (Bagnasco et al. 2022; Morey et al. 2021; Steven et al. 2021, 2014; Tella et al. 2015). Experiential learning from practice involves acquiring knowledge and skills through direct clinical experience and reflection. Kolb (2014) describes it as a process where learning emerges from concrete experiences. In nursing education, this approach effectively links theory to practice and enhances student motivation and engagement (Association of Experiential Education 2012; Kong 2021; Gross and Rutland 2017). Raising students' awareness of the concept of ‘safety culture’ may broaden their views of patient safety, help them recognise safety threats and enhance their overall knowledge and skills (Ramírez‐Torres et al. 2023; Bedgood and Mellot 2021). Adverse events and other patient safety incidents are used as an indicator of PSC, offering insights from students who have not yet fully integrated into it. While adverse events represent only one aspect of safety culture, they provide a valuable entry point for understanding how future healthcare professionals perceive and engage with safety principles. Research suggests that students' exposure to adverse events offers meaningful insights into PSC (Venesoja et al. 2022).

Against this backdrop, this study specifically aimed to explore Czech undergraduate nursing students' perceptions of PSC during clinical practice through a mixed‐method sequential study, providing insights into how these students experience and understand PSC in real‐world settings. The study was guided by the following research question: How do Czech undergraduate nursing students perceive PSC during their clinical practice?

## 3 Methods

### 3.1 Design

**FIGURE 1:**
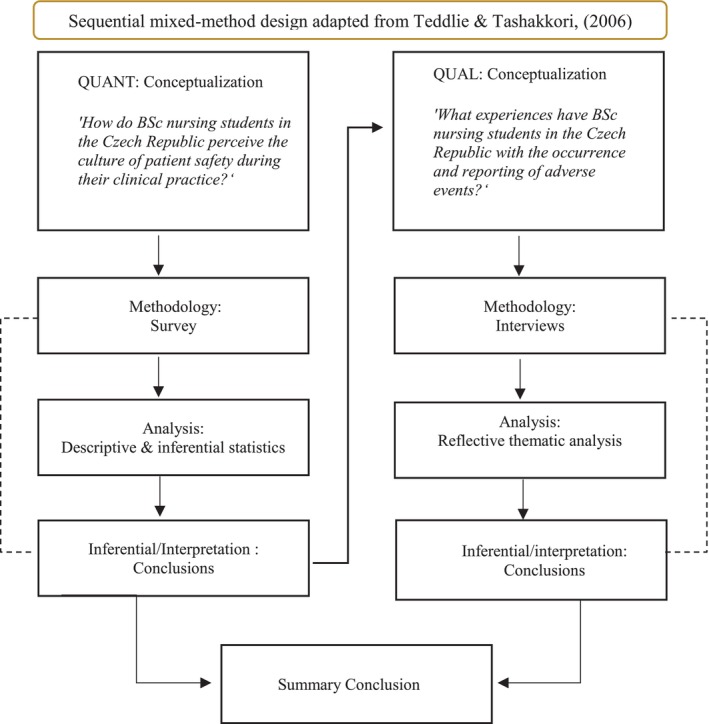
Methodological procedure of sequential mixed‐method design. QUANT, quantitative phase of the study; QUAL, qualitative phase of the study. [Colour figure can be viewed at wileyonlinelibrary.com]

A mixed‐method design was used to enable a multi‐faceted investigation that a single‐method approach could not provide (Creswell and Plano Clark 2017). In a sequential mixed‐method design, distinct phases occur chronologically, with each phase informing the next. In this study, findings from the initial quantitative phase shaped the research objectives for the subsequent qualitative component. The methodology followed nine structured phases (see Figure 1; Teddlie and Tashakkori 2006). Each phase adhered to the guidelines provided in the STROBE (von Elm et al. 2008) and COREQ (Tong et al. 2007) checklists.

### 3.2 Participants and Setting

In *the quantitative phase*, participants were selected through purposive sampling and included nursing students enrolled in the Bachelor of Science (BSc) Nursing programme across all 16 Czech faculties. Inclusion criteria required participants to be currently enrolled in the BSc programme and to have completed at least one clinical placement in a hospital setting. Students who had not yet undergone any hospital‐based clinical training were excluded. Faculty leaders distributed the survey link through class emails. To determine the appropriate sample size, an online calculator (Qualtrics 2020) was used, setting the minimum size at 346 respondents based on a 95% confidence interval and ± 5% margin of error, reflecting the 2016 population of 3456 nursing students in the Czech Republic (Institute of Health Information and Statistics of the Czech Republic 2017).

In *the qualitative phase*, purposive sampling was again used, this time targeting third‐year BSc Nursing students from one Czech faculty, irrespective of whether they were enrolled full‐time or part‐time. Inclusion criteria required that participants had experienced or directly observed one or more adverse events during clinical practice. Students without such experience were excluded. The decision to focus on third‐year students was based on the assumption that, by this stage in their programme, they would have accumulated a broader range of clinical experiences and greater exposure to patient safety issues, thus offering deeper insights relevant to the study's objectives. The head of the nursing department facilitated recruitment. The final sample size was based on the depth and complexity of information relevant to the study's objectives, as well as the researcher's critical appraisal; rather than reaching a saturation point, the data no longer provided new information, codes, or themes (Braun and Clarke 2021a).

### 3.3 Data Collection

Quantitative data collection took place between January and October 2021 using the Hospital Survey on Patient Safety Culture for Nursing Students (HSOPS‐NS), designed to gather nursing students' perceptions of PSC (de Ortiz Elguea et al. 2019). The HSOPS‐NS was translated and validated for use in the Czech language, addressing face, content and construct validity as well as reliability, with a high internal consistency (*α* = 0.914; Bartoníčková et al. 2024). The survey was distributed online through Google Forms following CHERRIES standards (Eysenbach 2004). Initial information on the web‐based survey concerned informed consent, and only after the student agreed were they redirected to the instrument.

The HSOPS‐NS consists of 54 items in four sections (A–D). The core of the instrument includes 49 items that gather nursing students' perceptions about a particular workplace, the evaluation of PSC in the hospital, communication in the workplace and perceptions of patient safety in general. Five additional items address the overall ‘patient safety grade’: the number of adverse events reported in the workplace (overall) and by nursing students (or under supervision), awareness of reporting systems, and space for additional comments on patient safety, errors, or reporting systems. Responses are scored on a 5‐point Likert scale (‘strongly disagree’ to ‘strongly agree’), with additional items scored on a 10‐point Likert scale or as dichotomous responses. At the end of the tool, sociodemographic data, including age, year of study, type of clinical placement and clinical supervision (mentor, nurse without specific training in mentorship, faculty staff member) were collected.

In the qualitative phase, data were collected from November 2023 to May 2024 through in‐depth, semi‐structured interviews using a topic guide developed from the quantitative results and refined with two experts (a qualitative researcher and a psychologist). Interview questions were pre‐tested with two potential participants (excluded from the main study) to ensure clarity and relevance. No modifications to the interview guide were necessary following the pretest.

Participants received detailed information regarding the study's objectives and scope before the interview. Face‐to‐face interviews were conducted in a private faculty office, audio‐recorded and lasted an average of 34 min (range 31:46–52:16). The interview guide focused on the dimensions rated lowest in the quantitative phase, covering topics such as the prevalence of adverse events, reasons for adverse events, prevention strategies and adverse event reporting. The term ‘adverse events’ is used throughout the findings to refer broadly to both harmful events and near misses or circumstances with the potential to cause harm. The interview guide also explored participants' experiences with the inclusion of adverse events in their education, such as whether, and in which courses, the topic was taught. Demographic data were also gathered.

In response to potentially distressing experiences related to adverse events, supervisors conducted debriefing sessions with students. These sessions were designed to provide emotional support, address concerns and facilitate reflective discussions in a safe and constructive environment.

### 3.4 Data Analysis

Data in the quantitative phase were analysed using IBM SPSS Statistics 25.0, employing both descriptive and inferential statistics. The missing data ranged from 0.2% to 0.4%. Descriptive statistics, including mean, standard deviation and frequency, were used to summarise the data. Non‐parametric tests were used based on the Kolmogorov–Smirnov test (0.057; *p* > 0.05). Multiple regression analysis identified predictors of overall patient safety grade, the number of events reported (overall) and the number of events reported by nursing students. Statistical significance was set at a *p*‐value of ≤ 0.05.

The qualitative data were transcribed verbatim and analysed through reflective thematic analysis drawing on Braun and Clarke's (2021b) approach, facilitated by NVivo software. This approach emphasises researcher subjectivity and reflexivity, with a focus on understanding rather than coding reliability. The analysis involved six stages: familiarisation, coding, generation of initial themes, review and specification of themes, final definition of themes and summary. To maintain the reflective analysis integrity (Braun and Clarke 2019), the line‐by‐line coding process was conducted by a single researcher, based on which first descriptive and analytic themes were created aligning with a critical realist epistemology (Archer et al. 2021). During this process reflective memos were kept which informed ongoing team discussions. Two experts (KŽ, SDC) reviewed and refined themes, checking for consistency and diversity in coding. During this process, some codes were consolidated into broader categories, and an additional theme was created (see File S1: Table S1). The final analytic themes and subthemes were based on 191 statement extracts organised into 107 codes, reflecting nursing students' experiences with adverse events. The process used triangulation of researchers and peer debriefing to ensure rigour and credibility. To enhance credibility, a qualitative research expert (KŽ) and an academic with a qualification in psychology (SDC) were involved in the peer review process during the four phases of reflective thematic analysis (Phases 3, 4, 5 and 6).

Although the phases were conducted sequentially, the study aimed to integrate the data types to form a cohesive and multi‐faceted understanding of the research problem. This integration was achieved through an explanatory design, where the results from one phase informed the next, connecting the data types. Triangulation and iterative thematic synthesis were employed to systematically link the phases, ensuring that the findings complemented and enriched one another (Creswell and Plano Clark 2017).

### 3.5 Ethical Considerations

Ethical approval was granted by the Ethics Committee (‘REDACTED’). Participation was voluntary with informed consent a prerequisite. Participants were assured their involvement would have no impact on their academic status or clinical practice. They were also informed of their right to withdraw from the study at any time without consequences. To protect anonymity in the qualitative phase, all interview participants were assigned pseudonyms when presenting the findings, making it impossible to identify which individuals were specifically included in the analysis.

## 4 Results—Quantitative

The sample consisted of 428 undergraduate nursing students. The mean age was 25.06 ± 8.05 years, with a range of 18–57 years. Sample characteristics are reported in Table 1.

**TABLE 1:** Sociodemographic variables (quantitative).

Sociodemographic variable	*n*	%
Year of study		
1st	169	35.1%
2nd	169	35.1%
3rd	144	29.9%
Form of study		
Full‐time	270	56.0%
Part‐time	212	44.0%
Type of clinical placement		
Outpatient care^a^	60	12.4%
Medical–surgical inpatient care	296	61.4%
Critical or intensive care^b^	71	14.7%
Mother–child inpatient care^c^	34	7.1%
Other areas	21	4.4%
Clinical supervision		
Faculty staff member	53	11.0%
Mentor	101	21.0%
Nurse without specific training in mentorship	328	68.0%

^a^ Day clinics, primary care and rehabilitation.

^b^ Critical‐special services, intensive care, accident and emergency and the OR.

^c^ Maternity and paediatrics, obstetrics, gynaecology.

### 4.1 Patient Safety Culture Perceived by Nursing Students During Clinical Practice

The results of the evaluation for each dimension of PSC are summarised in Table 2. The dimensions with the lowest ratings were Frequency of Events Reported (37.0%) and Nonpunitive Responses to Errors (42.4%).

**TABLE 2:** Evaluation of PSC dimensions by nursing students.

Dimensions of PSC	M ± SD	%*
Teamwork within units	3.88 ± 0.77	70.4
Supervisor/manager expectations and actions promoting patient safety	3.79 ± 0.80	65.2
Organisational learning/continuous improvement	3.55 ± 0.72	56.0
Management support for patient safety	3.66 ± 0.84	62.7
Overall perceptions of patient safety	3.82 ± 0.71	66.7
Feedback and communication about error	3.63 ± 0.79	61.5
Communication openness	3.82 ± 0.80	69.5
Frequency of events reported	3.20 ± 0.90	37.0
Teamwork across units	3.45 ± 0.70	52.5
Staffing	3.31 ± 0.73	45.9
Handoffs and transitions	3.73 ± 0.72	62.7
Nonpunitive responses to errors	3.30 ± 0.78	42.4
Indicator of good praxis	3.62 ± 0.70	54.1

Abbreviations: M, Mean; PSC, patient safety culture; SD, Standard Deviation.

* Percentage of positive responses (Agree/Strongly Agree).

The overall patient safety grade was rated at 7.81 ± 1.54 on a scale from 1 to 10. On average, students reported 2.39 ± 3.88 adverse events overall occurring in the workplace over the past 12 months, with some reporting as many as 20 events. However, the mean number of events directly reported by nursing students was only 1.15 ± 5.04 (range 0 to 20). While 60% of students were aware of the adverse event reporting system, 40% lacked this awareness.

### 4.2 Predictors of the Overall Patient Safety Grade, the Number of Reported Events and the Number of Reported Events by Nursing Students

Model 1 (*R* ^2^ = 0.302; Adj. *R* ^2^ = 0.282; *F* = 15.567; *p* < 0.001) identified that five dimensions of the PSC accounted for 28.2% of the variance in the overall patient safety grade. Model 2 (*R* ^2^ = 0.057; Adj. *R* ^2^ = 0.030; *F* = 2.157; *p* = 0.010) showed a significant relationship between two dimensions of PSC and the number of events reported in the workplace, explaining 3% of the variance. Model 3 (*R* ^2^ = 0.055; Adj. *R* ^2^ = 0.028; *F* = 2.071; *p* = 0.015) found that three dimensions of PSC explained 2.8% of the variance in the number of events reported by nursing students. The results are presented in Table 3.

**TABLE 3:** Predictors of patient safety grade, number of events reported, number of events reported by nursing students.

PSC dimensions (Independent variables)	Dependent variables					
	Overall patient safety grade		Number of events reported (overall)		Number of events reported by NS	
	*β*	*p*	*β*	*p*	*β*	*p*
(Constant)	0.001**		0.042*		0.142	
Teamwork within units	0.090	0.103	−0.053	0.413	−0.159	0.014*
Supervisor/manager expectations and actions promoting patient safety	−0.055	0.323	−0.059	0.358	0.018	0.777
Organisational learning/continuous improvement	0.061	0.250	0.093	0.133	0.095	0.125
Management support for patient safety	0.135	0.007*	−0.011	0.854	−0.007	0.909
Overall perceptions of patient safety	0.148	0.006*	−0.091	0.143	−0.020	0.750
Feedback and communication about error	0.112	0.049*	0.021	0.752	−0.055	0.402
Communication openness	−0.026	0.619	0.082	0.181	−0.008	0.901
Frequency of events reported	0.031	0.460	0.004	0.942	−0.024	0.624
Teamwork across units	0.110	0.027*	−0.068	0.239	−0.004	0.942
Staffing	0.121	0.006*	−0.106	0.039*	−0.030	0.556
Handoffs and transitions	0.084	0.107	−0.047	0.435	−0.144	0.018*
Nonpunitive responses to errors	0.063	0.158	0.075	0.145	0.069	0.182
Indicator of good praxis	−0.017	0.728	0.139	0.018*	0.204	0.001**

*Note:* β = Standardised regression coefficient; Bold values, *p* = *p*‐value: *p ≤ 0.05; **p ≤ 0.001. All displayed variables were included in the regression model. Abbreviation: PSC, patient safety culture.

## 5 Results—Qualitative

The sample included 12 third‐year BSc Nursing students. The mean age was 24.17 years, with a range from 21 to 42 years, with others reported in Table 4.

**TABLE 4:** Sociodemographic variables (qualitative).

Participant	Age	Form of study	Type of clinical placement, where the adverse event occurred	Clinical supervision
Emily	22	Full‐time	Neurology	Mentor
Mia	22	Full‐time	Long‐term care hospital	Nurse without specific training in mentorship
Olivia	21	Full‐time	Neurosurgery	Mentor
Sarah	42	Part‐time	Medical inpatient care	Faculty staff member
Valentina	21	Full‐time	Medical inpatient care	Mentor
Nora	21	Full‐time	Medical inpatient care	Mentor
Karen	22	Full‐time	Medical inpatient care	Mentor
David	25	Part‐time	Medical inpatient care	Faculty staff member
Andrea	23	Full‐time	Traumatology	Mentor
Nina	22	Full‐time	Medical inpatient care	Mentor
Michael	21	Full‐time	Medical inpatient care	Mentor
Oliver	28	Part‐time	Surgical inpatient care	Mentor

### 5.1 Nursing Students' Experience of Adverse Events During Clinical Practice

**FIGURE 2:**
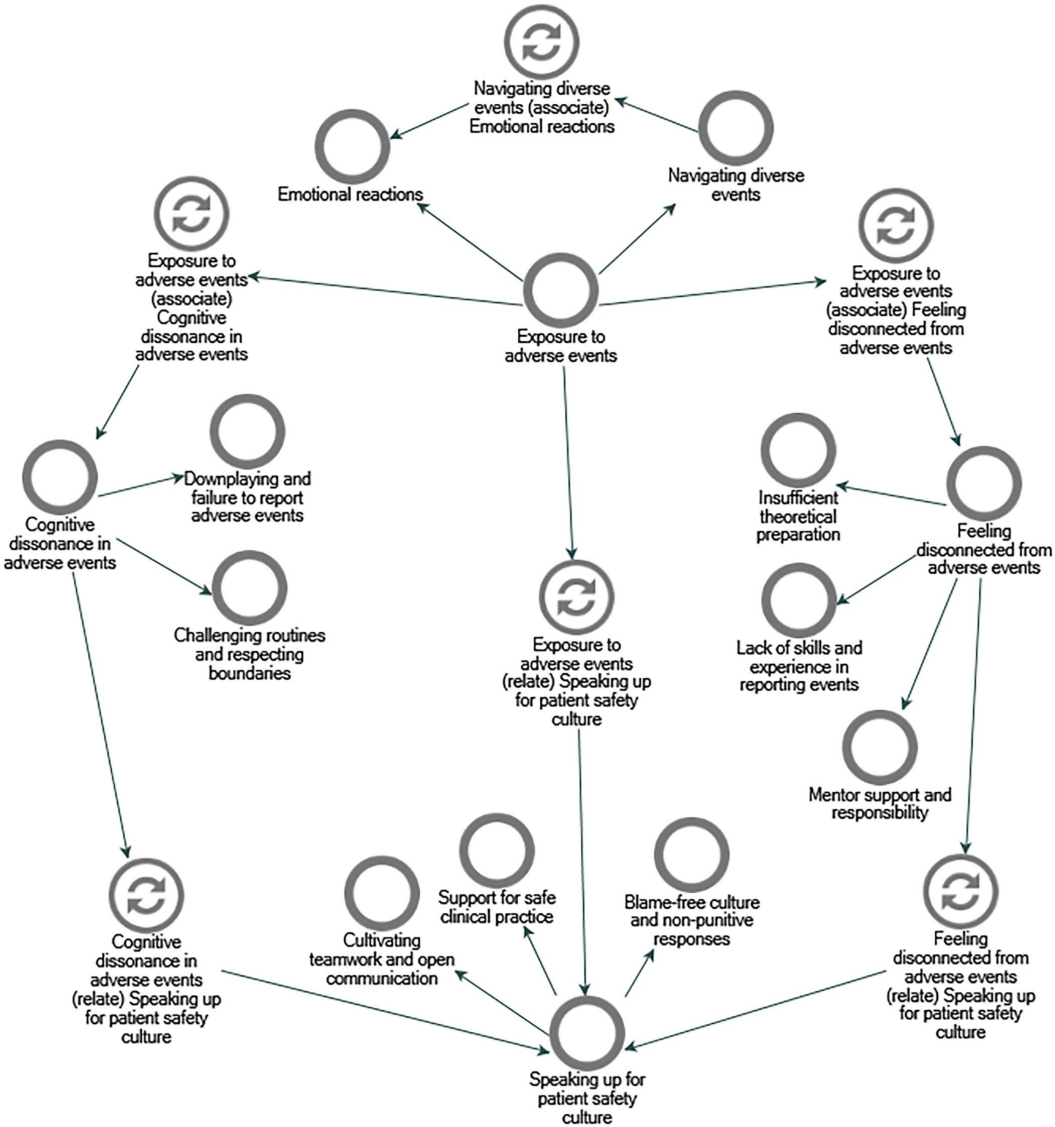
Nursing students' experience of adverse events: themes and subthemes. [Colour figure can be viewed at wileyonlinelibrary.com]

The reflective journey of nursing students' experiences with adverse events in clinical practice is embodied in 4 main themes and 10 subthemes (Figure 2). The journey reflects a progression from an initial *Exposure to Adverse Events*, leading to *Feeling disconnected* and *Cognitive Dissonance*, through to a culmination via *Speaking Up for Patient Safety Culture* as a shared goal to prevent adverse events in clinical settings.

## 6 Theme 1: Exposure to Adverse Events

The encounters students had with events began as an ‘exposure’ which stimulated a range of emotions.

### 6.1 Navigating Diverse Events

Analysis of the nursing students' accounts revealed diverse types of adverse events encountered in clinical practice. Students were directly involved in some events and observers of others. The recurring patterns in their narratives highlighted patient falls and pressure ulcers as the most frequently encountered events:

… the most common was probably patient falls and also pressure ulcers. (Olivia)

Medication errors emerged as another prominent issue, often involving mix‐ups with medication or patient identity. Although students often found themselves in an observer role, sometimes they were personally involved:

The nurse grabbed the wrong end of the infusion and was supposed to give one infusion to the other patient, but she almost administered it to the wrong one. (Nora)

It happened to me personally that I almost mixed up the medication, but I realized it in time and corrected it. (Mia)

In addition to these common incidents, students recounted rarer but more severe events, such as transfusion errors, a patient bed crashing through a glass door and patients missing from the ward. Certain adverse events, like needle and ampule injuries, posed direct harm to the students themselves:

…like when I burst the ampule, I cut myself and then pricked myself while diluting the medication. (Valentina)

These reflections demonstrate nursing students encounters with adverse events in clinical practice, highlighting both the frequency and intensity of such experiences in their learning journey.

### 6.2 Emotional Reactions

When nursing students encountered an adverse event, they experienced a range of predominantly negative emotional reactions. Fear emerged most frequently, often surfacing immediately after the event and tied to concerns about the patient's health and potential repercussions within the nursing team:

I was worried because I knew it would cause trouble if it was documented… I knew the head nurse would address it. (Michael)

Where students were involved in the event, their emotions were directed inwards at themselves:

It was an absolute shock for me… I completely froze. It felt like my own failure. (Sarah)

I was so disappointed in myself, feeling like I wasn't as good of a nurse or student as I should be (Mia)

These emotional reactions highlighted the weight nursing students feel when adverse events occur, reflecting both personal accountability and the pressure to maintain patient safety.

## 7 Theme 2: Feeling Disconnected From Adverse Events

Participants described a sense of disconnect from the events they experienced or witnessed. This appeared related to gaps in their preparation or experience, which were sometimes exacerbated by supervision and mentoring issues.

### 7.1 Insufficient Theoretical Preparation

Nursing students often linked events and a sense of disconnect as arising in part from gaps in their knowledge stemming from insufficient theoretical preparation during their studies:

We don't know if things like slippery floors or the use of bed rails make sense in preventing adverse events. (Andrea)

In the BSc Nursing programme, adverse events were typically covered indirectly within other subjects, leading students to perceive the topic as underemphasised and inadequately addressed:

It's a topic we only touched on in a few subjects, but it wasn't a stand‐alone subject. (Nina)

These comments indicated a perceived need for education focused specifically on adverse events to better prepare nursing students for clinical practice. The implication was that such preparation would enable them to feel ‘closer’ to an event, either through helping avoid it or dealing with the aftermath, through greater understanding.

### 7.2 Lack of Skills and Experience in Reporting Events

Students also attributed many adverse events to their own inadequate clinical skills, which were related to feelings of uncertainty, rashness and inexperience:

The student will make that mistake anyway in the vast majority of cases out of some hastiness or uncertainty, indiscretion, lack of practice. (Oliver)

Another key issue highlighted by participants was a lack of knowledge and experience in event reporting. Many had never been involved in the reporting process or received training on the systems used, leaving them unsure how to document such events in a clinical setting:

I've never really dealt with it. I've never been part of an adverse event report. I don't know how it should be recorded. (Michael)

These experiences highlight the need for practice‐based training on patient safety event reporting to prepare nursing students to effectively manage and document such events in clinical practice.

### 7.3 Mentor Support and Responsibility

In clinical practice, nursing students rely on mentor supervision and often view mentors as crucial for guidance and for safeguarding against adverse events:

The student works under the nurse supervision there anyway, so [mentor]should consult with [them: student] about everything. And supervise [student] basically…. (Emily)

However, mentors often assigned students responsibilities and autonomy that exceeded their level of preparedness, assuming they had sufficient theoretical knowledge:

It's just that the nurse, like who's in control of them, maybe misjudges somehow their sort of ability and knowledge and just gives them like a big slice of bread, so to speak, that they're not able to deal with. (Michael)

Students often expressed uncertainty about their own independence and attributed adverse events to a lack of supervision. This uncertainty, coupled with fear, stemmed from a reluctance to ask for direction and a fear that mistakes would further undermine their confidence:

The nurses should have taken into account that the student is still just a student. [They are] just learning. And not just blame it on [student]. (Nina)

These considerations emphasised the need for a balanced approach to mentor supervision, ensuring that students felt supported while being encouraged to develop new skills and gain experience, thereby boosting their self‐confidence—without being overwhelmed by clinical responsibilities.

## 8 Theme 3: Cognitive Dissonance

Students often seemed to experience a sense of internal conflict in relation to patient safety events.

### 8.1 Challenging Routines and Respecting Boundaries

Participants felt the causes of adverse events often related to nurses' failure to meet standards and sometimes lacked competence. From their own learning, students identified some practice routines they witnessed as incorrect:

Most of those nurses who have been in practice for a long time just have their routine and don't address whether it's right. (Nina)

Students noted that both nurses and some peers often worked beyond their competency level or scope of practice. This issue was compounded by an expectation that students, regardless of their level of education, would undertake roles typically reserved for qualified nurses:

Simply, in practice, no one asks if you are a student or a nurse. You're just a nurse, and you're just going to do what a nurse does. And if you try to say it's not in your scope of practice, nobody cares. (David)

These statements highlighted the importance of adherence to standards and competencies to prevent adverse events and support student growth within boundaries.

### 8.2 Downplaying and Failure to Report Events

Students reported that adverse events were often underestimated or downplayed by nurses:

The nurse basically just waved her hand over it, completely downplaying anything that happened. (Nora)

They noticed that many nurses avoided formally reporting adverse events, often hiding them for fear of repercussions:

Most adverse events stay hidden because some nurses don't want to report them and get in trouble. (Olivia)

When uncertain of the potential outcome, nurses were seen to sometimes consult senior staff about the seriousness of the error, yet reporting was rarely observed:

If there's a medication error, nurses ask the doctor if it's serious, but I've never seen anyone actually write it down. (Andrea)

These experiences emphasise the need for encouragement of reporting and development of a learning culture where adverse events are not concealed or downplayed.

## 9 Theme 4: Speaking Up for Patient Safety Culture

Students reflected on raising concerns about patient safety, identifying what they viewed as key issues.

### 9.1 Cultivating Teamwork and Open Communication

Students identified teamwork and open communication as key elements in preventing adverse events. They noted that strong teamwork could mitigate events, while poor collaboration could contribute to them. A need for continuous improvement in teamwork was also emphasised, for example, through regular team sessions focused on adverse events to foster a culture of support and responsibility:

I think the cooperation in that team is very important. (Andrea)

Open communication about adverse events was highlighted as essential. Students felt that speaking up about adverse events and learning from these experiences should be an integral part of the nursing curriculum. The openness of communication promotes proactive learning and helps students and staff identify and deal with problematic areas, which leads to improved patient safety:

It's important to highlight problem areas to students and how to prevent them because that's where it all starts. (Mia)

These insights reflected the need for a more collaborative and communicative environment where teamwork and openness are actively encouraged, creating a foundation for safer clinical practice.

### 9.2 Support for Safe Clinical Practice

Nursing students emphasised that preventing adverse events requires a strong foundation of management support, continuous learning and adequate staffing. They identified the active involvement of management as key, playing a role in motivating staff, maintaining adequate staffing levels and promoting adherence to safety standards. This creates a controlled, well‐structured and safe environment that enhances patient safety as well as student learning:

…it's definitely in the management actually, in terms of the head nurse or the overall management of the health facility. (Karen)

Adequate staffing was highlighted as a crucial element in minimising adverse events and creating a supportive environment. Students highlighted the importance of retention of staff and support for nursing students during clinical practice to ensure a stable workforce engaged in safe practice:

…and when they have staff, keep them, and treat students on internships so they want to stay. (Sarah)

Finally, students also emphasised the importance of continuous learning in the prevention of adverse events:

Just like the annual hand hygiene training, I think it wouldn't hurt anyone to have an hour once a year to simply talk about this. (David)

This combined approach of management support, continuous learning and adequate staffing forms a comprehensive strategy to promote a safe clinical environment that benefits patients, nurses and nursing students.

### 9.3 Blame‐Free Culture and Nonpunitive Responses

A nonpunitive environment is essential for nursing students to cope with adverse events. Blaming and stigmatisation discourage adverse event reporting, while a focus on learning from mistakes—both one's own and others'—promotes transparency and improvement. This attitude allows for the development of effective strategies to prevent mistakes from being repeated:

The greatest lesson is learned when you see something happen to someone else, think about whether it could happen to you, and realize you must avoid it. (Oliver)

A blame‐free culture based on the shared experiences of nursing students encourages open communication without punishment and proactive learning from adverse events.

## 10 Discussion

The study aimed to investigate Czech undergraduate nursing students' perceptions of PSCs during clinical practice, with a particular focus on experience of adverse events and their management. A sequential mixed‐methods study design was used (Creswell and Plano Clark 2017). This design has seldom been applied to patient safety research with nursing students (e.g., Çatal et al. 2024; Sinclair et al. 2016; Roney et al. 2017), despite the potential added insights it may offer (Creswell and Plano Clark 2017). Limited use may stem from the complexity, resource demands and logistical challenges of coordinating multi‐phase data collection in clinical settings. This study provides novel insights into students' perceptions and experiences of patient safety in clinical practice, utilising a seldom employed methodology to contribute to the existing knowledge base.

The most negatively rated dimension of PSC in this study related to adverse events and comprised both the frequency of events and responses to them. Globally, adverse events are one of the worst‐rated dimensions in terms of patient safety (e.g., Duhn et al. 2012; Suliman 2019; Usher et al. 2017). The most common adverse events encountered by nursing students in clinical practice—such as falls, pressure ulcers, medication errors and sharps injuries—were also identified in a study conducted in Slovenia by Gradišnik et al. (2024). Students in such situations express various emotions, with fear being dominant, especially in relation to potential consequences, but also, according to Jones et al. (2013), in terms of concerns regarding authority. Deeper and prolonged emotional experiences tend to be associated with the type of adverse event and may result in personal harm (Gradišnik et al. 2024), somatic symptoms, confusion, or depression (Kang and Cho 2023). Thus, adverse events have implications for patients, staff, healthcare systems and also students (Liukka et al. 2020).

Students' attitudes toward adverse events are significantly correlated with perceptions of safety climate, professional identity and negative feelings about the transition to qualified nursing practice (Tao et al. 2023). In our study, adverse event reporting, both overall and at the level of individual nursing students, was predicted by the ‘Indicators of Good Practice’ dimension from the HSOPS‐NS questionnaire. These findings were further reflected in the qualitative phase, where students experienced cognitive dissonance, particularly when witnessing non‐adherence to standards and competencies in clinical practice. Cognitive dissonance is a state of tension between beliefs and behaviours observed or engaged in, and can result in emotional distress (Cooper and Carlsmith 2015). Such dissonance is often evident where there is inconsistency between what students learn in the academic settings and what they confront in practice (Fagan et al. 2021), and where they sense a lack of emotional safety in the learning environment (Steven et al. 2023, 2014). Feeg et al. (2021) highlight that nursing students frequently encounter ethical dilemmas in clinical practice that conflict with their professional values or theoretical knowledge, leading to moral distress and cognitive dissonance. These moments, while emotionally challenging, can prompt critical reflection and foster ethical awareness and professional identity development when supported appropriately within the educational setting.

Various factors can influence nursing students' willingness to speak up, with defensiveness by clinical staff acting as an inhibitor (Ghasempour et al. 2023). Fagan et al. (2021) also highlight how insufficient theoretical preparation can make students hesitant to voice concerns, as it leaves them feeling unprepared to effectively address or articulate issues. This aligns with the sense of disconnect experienced by students in this study. Exposure to these situations is often referred to as the ‘context of disclosure’ (Fisher and Kiernan 2019). Underreporting, as well as downplaying adverse events, exacerbates cognitive dissonance. As a result, students may deny or fail to recognise an incident as an adverse event, sometimes resorting to concealment or dishonesty (Ghasempour et al. 2023). Reporting adverse events is often a sensitive or avoided topic among students, as they may hesitate to discuss it openly due to fear of judgement, lack of confidence, or potential repercussions (e.g., Gunes et al. 2020; Sepp 2024). Notwithstanding the strength of this dissonance, the power of learning through observing practice and the influence of clinical social norms, often results in the formation of suboptimal routine standards (Ryan et al. 2021). Furthermore, developing low vigilance and adopting suboptimal practice can negatively impact students and their patients in their future professional lives (Fagan et al. 2021; Kang and Cho 2023).

In the Czech Republic, up to 40% of nursing students reported being unaware of systems for reporting adverse events. Similarly, Ramírez‐Torres et al. (2023) found that a significant proportion of students either did not report or only occasionally reported patient safety concerns. This aligns with earlier research by Palese et al. (2018) and Stevanin et al. (2018), which documented limited reporting behaviours among nursing students. This reluctance is often driven by a strong desire to survive clinical practice and complete their placements (Fisher and Kiernan 2019), coupled with barriers such as fear of repercussions, lack of awareness and unprofessional workplace behaviours (Halperin and Bronshtein 2019; Li et al. 2021). Additionally, students often perceive disengagement within the team and nurses' dismissal of adverse events in clinical practice, which can reinforce silence and non‐reporting. This is compounded by the fact that most nursing curricula do not include formal training in reporting procedures or systems‐based responses to errors, leaving students underprepared for this aspect of patient safety. To address this, nursing education should integrate targeted strategies—such as simulation, open discussion of errors and mentoring—to foster a supportive culture where reporting is viewed as a professional responsibility (Kang and Cho 2023).

A lack of theoretical preparation can further hinder students' willingness to speak up, as it leaves them feeling unprepared to address safety concerns effectively (Fagan et al. 2021). The disconnect between academic preparation and clinical reality, often described as the ‘theory‐practice gap’, contributes to a reality shock when students encounter the demands of clinical practice (Lundell Rudberg et al. 2022; Kyrkjebø and Hage 2005). Academic education sometimes prioritises instrumental nursing procedures over patient safety concepts, reducing the visibility of patient safety in the curriculum (Boucaut and Cusack 2016; Vaismoradi et al. 2011; Ryan et al. 2021; Steven et al. 2014; Kirwan et al. 2019). This aligns with findings by Levett‐Jones and colleagues, who emphasise that confronting poor practices and demonstrating moral courage are critical for nursing students' professional growth (Bickhoff et al. 2016; Jack et al. 2020, 2021).

Mentors play a pivotal role as bridges between academia and clinical practice, offering guidance and modelling appropriate behaviours that support students' development (Steven et al. 2014; Javornická et al. 2024; Lundell Rudberg et al. 2022). However, when adverse event reporting is neglected by clinical staff or justified as non‐consequential, students may internalise these behaviours (Fisher and Kiernan 2019; Atakro et al. 2019). Gradišnik et al. (2024) describe how students' non‐reporting is often influenced by a hope that no harm will occur to patients or themselves. Emotional learning, fostered through exposure to such situations, is critical for professional development and should reflect the highest standards, including regular reporting of adverse events, regardless of the perceived risk to patients (Espin and Meikle 2014; Steven et al. 2023; Venesoja et al. 2022). In the Czech Republic, specific training programmes for clinical mentors have been developed to improve their ability to support students in clinical learning. However, participation in these programmes is not mandatory, which may contribute to variability in mentorship quality. In addition to strengthening access to structured mentor training, it could be useful to implement formal feedback mechanisms, reflective supervision sessions and peer‐mentoring models to enhance students' confidence and ability to speak up in clinical environments.

The factors influencing the overall grade of patient safety were identified as the influence of management, overall perception of safety, feedback and communication, teamwork and staffing. These aspects were further substantially reflected in the qualitative phase of the research, which served as the foundation for the outcomes. Students placed great importance on teamwork and communication, perhaps recognising the important role these play in speaking about patient safety (Fisher and Kiernan 2019), adverse events (Ryan et al. 2021; Venesoja et al. 2022) and the recovery process (Zieber and Williams 2015). In terms of open communication, feedback is especially important (Lyman and Mendon 2021) and should be based on a blame‐free and nonpunitive culture. Learning from mistakes through situational learning can contribute to greater student cautiousness (Gradišnik et al. 2024) and the development of emotional safety mechanisms in clinical practice (Steven et al. 2023). Support for clinical practice then relies primarily on management accountability, continuous learning and staffing. Management may significantly influence these aspects once students are convinced of its effectiveness (Hood and Copeland 2024).

The findings of this study are also important in light of Bazrafshan et al. (2025), who demonstrated that nursing students' experiences with clinical errors represent meaningful opportunities for experiential learning, contributing to their understanding of patient safety and their ability to manage complex situations. This aligns with Kolb's theory (2014) and supports prior research showing that real‐life experience fosters professional development and adaptability (Gross and Rutland 2017; Kong 2021; Association of Experiential Education 2012). However, the results also highlight persistent gaps in theoretical preparation regarding patient safety, suggesting that the culture of safety is not sufficiently integrated into current nursing curricula. To address this, educational programmes should consider incorporating structured reflection sessions following clinical experiences, simulation‐based learning focused on error prevention and management and dedicated coursework on patient safety principles and systems thinking. Embedding these components systematically into the curriculum could enhance students' readiness to practice safely and foster a deeper understanding of safety culture as an integral part of professional nursing identity.

### 10.1 Limitations

A limitation of using a mixed sequential study design is the increased risk of bias, as the results of the first phase can overly influence the interpretation of the second. However, it can be argued that although the phases were chronological, they were not separate but interrelated. To enhance analysis and reflexivity by bringing other perspectives to bear on the data, experts were invited to review and ‘validate’ the candidate themes identified in the qualitative phase.

The use of self‐report tools offers some anonymity but may still be influenced by unconscious negative evaluation, for example, due to the emotions of the students at the time of completion. However, it was partly these emotional responses we were interested in as they form a key part of the students experience. Social desirability can impact face‐to‐face data collection; therefore, to mitigate this, both self‐reports and interviews were used to provide a balanced approach to data collection.

In the qualitative phase, the inclusion of students from only one faculty may be considered a limitation potentially impacting on the generalisability of the findings. However, the homogeneity of the sample was a deliberate methodological choice to allow for in‐depth exploration of shared experiences within a specific educational and clinical context. While the results cannot be generalised to all nursing students in the Czech Republic or beyond, the consistency of our findings with those reported in previous international studies suggests that the core issues identified may have a degree of transferability to similar settings.

## 11 Conclusion

Nursing faculties are strategically positioned to ensure that patient safety, with a strong focus on adverse events, is thoroughly integrated into the curriculum for BSc nursing students. By embedding these principles into their education, the quality and safety of nursing care can be significantly enhanced. During clinical practice, nursing students encounter various unsafe practices, which can shape their future professional behaviours. Indicators of good practice are essential for promoting the reporting of adverse events, while the feeling of being disconnected from such events and the potential for cognitive dissonance must be addressed. Fostering a strong PSC and emotionally safe learning environment within BSc programmes and clinical areas is a crucial foundation for achieving these goals. With regular assessments and targeted interventions, a safe and supportive environment can be created not only for nursing students but also for other healthcare professionals and, most importantly, for patients.

## Ethics Statement

Ethical approvals (UPOL‐109784/1050S‐2020; UPOL‐15475/FZV‐2023) were obtained from the Ethics Committee of the Faculty of Health Sciences at Palacký University Olomouc. Confidentiality and anonymity were maintained, and the research was carried out according to the recommendations of the Declaration of Helsinki.

## Conflicts of Interest

The authors declare no conflicts of interest.

## Supporting information


**Data S1.** COREQ.

**Data S2.** STROBE_checklist_2.

**Table S1.** Examples of the process of creating the themes and subthemes from significant statements.

## Data Availability

The data that support the findings of this study are available from the corresponding author upon reasonable request.
