# Supplementary material for: Exploring Nursing Students' Perspectives on Patient Safety Culture in Clinical Settings: A Mixed‐Method Study
Source: J Clin Nurs. 2025 May 6;34(7):2702–17. doi: 10.1111/jocn.17812 (PMC12181154; doi:10.1111/jocn.17812)
Supplement: Supplementary file 3 — Table S1. Examples of the process of creating the themes and subthemes from significant statements. [file JOCN-34-2702-s002.docx]

**Supplementary file. Table 1:** Examples of the process of creating the themes and subthemes from significant statements

| **Preliminary themes** | **Preliminary subthemes** | **Examples of statements** | **Final subthemes** | **Final themes** |
| --- | --- | --- | --- | --- |
| Encounter with adverse events | Types of adverse events | *“Well, in the morning during the morning hygiene routines, a gentleman went to the bathroom, and he happened to fall.”* (Emily) | Navigating diverse events | Clash with adverse events |
|  |  | *“My fault was that I misidentified the patient who should have been lying in the room in the first place. He was lying on the other bed, and I gave it to another patient.”* (Oliver) |  |  |
|  |  | *“For me, the worst was the transfusion mix-up, where the blood type was the same.”* (Sarah) |  |  |
|  | Nursing students' experiences | *“My greatest fear was with the gentleman as he came through the glass door and the delivery of the berodual.”* (David) | Emotional reactions |  |
|  |  | *“It was an absolute shock for me... It was like I stopped completely, and I froze... It felt like my own failure when the event happened”* (Mia) |  |  |
|  |  | *"I was just so scared, insecure, like for like a week afterwards I was thinking, you know, if it's going to do anything."* (Valentina) |  |  |
| Transition from theory to practice | Insufficient theory | *„What comes to my mind is that I know, for example, that some classmates from high school had problems during their first-year initial clinical practice.”* (Mia) | Insufficient theoretical preparation | Feeling disconnected from adverse evets |
|  |  | *We hadn’t learned nearly any procedures yet, so it was more about just getting familiar with the environment. And during the second clinical practice, my mentor was told that I was a practical nurse. She had no idea that I had just graduated from high school.* (Nina) |  |  |
|  |  | *"So maybe someone just mentioned it in passing, but I can't remember exactly which subjects it was in besides the procedures."* (Karen) |  |  |
|  | Insufficient experience | *"Hmm, maybe it's recorded both on paper and electronically, in some sort of book um in some sort of record of those adverse events I would say it's reported. Hmm, but then where exactly? I guess I don't know, unfortunately."* (Olivia) | Lack of skills and experience in reporting events |  |
|  |  | *“The nurse always has control over us.”* (Valentina) | Mentor support and responsibility |  |
|  |  | *“That was also a mistake that was partly caused, possibly because the mentor didn’t check the student’s work.”* (Oliver). |  |  |
|  | Handling events during practice | *“They didn’t really prepare the medications as they were supposed to… they prepared them in the nurse's station into pill organizers and then distributed them gradually.”* (Mia) | Challenging routines and respecting boundaries | Cognitive dissonance |
|  |  | *“Nowadays, it’s not uncommon for the work of registered nurses to be shifted onto practical nurses in some departments, and then the practical nurse might not know what or how to do certain things.”* (David) | Downplaying and failure to report events |  |
|  |  | *“The nurse didn’t report that she had accidentally pricked herself with a used needle while administering insulin to a patient. And she didn’t report it or even mention it to her colleagues.”* (Karen) |  |  |
| Importance of patient safety culture | Teamwork and collaboration | *“Surely, the overall atmosphere within the team also has an impact on it.”* (Karen) | Cultivating teamwork and open communication | Speaking up for patient safety culture |
|  |  | *"The staff should like work together somehow."* (Nina) |  |  |
|  | Openness in communication | *“If we talked about it, I think the others could gain a lot from it as well.”* (Michael) |  |  |
|  | Management support | *“The task of management is to establish a certain level or ensure adherence to standards. I think that as a leader, they should educate their subordinates about following these standards.”* (Mia) | Support for safe clinical practice |  |
|  | Staffing | *“It’s important to have more staff so that there is a greater sense of calm, and everyone knows the work will get done under less pressure.”* (Andrea) |  |  |
|  | Continuous learning | *“Important are educational events, like, I don’t know, some seminars on things like pressure ulcers, so that they know and have the knowledge necessary for prevention.”* (Olivia) |  |  |
|  | Blame free and non-punitive culture | *“The key is not to blame the staff for it and not to present it as a terrible problem for which we will be punished.”* (Michael) | Blame-free culture and non-punitive responses |  |
|  |  | *“We can learn from the mistakes of others.”* (Andrea) |  |  |
|  |  | *“It’s important to learn from previous events, build on them, or come up with different procedures.”* (David) |  |  |
